# Spectrum of TB Disease and Treatment Outcomes in a Mobile Community Based Active Case Finding Program in Yogyakarta Province, Indonesia

**DOI:** 10.3390/tropicalmed8090447

**Published:** 2023-09-15

**Authors:** Nur Rahmi Ananda, Rina Triasih, Bintari Dwihardiani, Betty Nababan, Arif Hidayat, Geoff Chan, Philipp du Cros

**Affiliations:** 1 Pulmonology Division, Department of Internal Medicine, Faculty of Medicine, Public Health and Nursing Universitas Gadjah Mada/Sardjjto Hospital, Sleman, Yogyakarta 55281, Indonesia; 2 Department of Pediatric, Faculty of Medicine, Public Health and Nursing, Gadjah Mada University/Dr. Sardjito Hospital, Sleman, Yogyakarta 55281, Indonesia; 3 Center of Tropical Medicine, Faculty of Medicine, Public Health and Nursing, Universitas Gadjah Mada, Yogyakarta 55281, Indonesia; 4 Tuberculosis Elimination and Implementation Science Group, Burnet Institute, Melbourne, VIC 3004, Australia; 5 Monash Infectious Diseases, Monash Health, Melbourne, Clayton, VIC 3168, Australia

**Keywords:** active case finding, subclinical TB disease, spectrum TB disease, treatment outcome

## Abstract

The World Health Organization recommends using chest X-ray (CXR) in active case finding (ACF) to improve case detection. This study aimed to describe the spectrum and outcomes of TB disease diagnosed through a mobile community based ACF program in Yogyakarta. This prospective cohort study included people attending a TB ACF program in Yogyakarta between 1 January 2021 to 30 June 2022. Participants ≥10 years old underwent CXR, symptom screening, and Xpert MTB/RIF testing of sputum. Subclinical TB was defined as asymptomatic active TB, whether bacteriologically confirmed or not. Treatment outcome data were obtained from the national program TB database. 47,735 people attended the ACF program; the yield of TB disease was 0.86% (393/45,938). There were 217 symptomatic cases, of whom 72 (33.2%) were bacteriologically confirmed, and 176 asymptomatic cases, with 52 (29.5%) bacteriologically confirmed. Treatment success was 70.7% with high loss to follow up (9%) and not evaluated (17.1%). Multivariate analysis demonstrated weak evidence for lower unsuccessful outcomes in symptomatic versus subclinical TB (aOR 0.6, 95% CI 0.36–0.998). TB ACF programs utilizing CXR may diagnose a high proportion of subclinical TB. Linkage to care in ACF program is important to increase successful treatment outcomes.

## 1. Introduction

Tuberculosis (TB) can be challenging to diagnose as active TB disease can occur without any reported symptoms and not all TB disease can be bacteriologically confirmed with current TB laboratory investigations [1,2,3]. The clinical spectrum of TB is a disease continuum divided into several manifestations: past eliminated TB infection, latent TB infection (dormant bacilli and inactive), incipient (viable *Mycobacterium tuberculosis*, likely to progress to active disease) [4], subclinical TB (no symptoms of TB but the bacilli are actively replicating), and active TB disease [5]. Detection of cases as early as possible is urgently needed to achieve TB elimination.

It is important to diagnose individuals with subclinical TB as they can contribute to TB transmission and early treatment could prevent development of health impacts and worse outcomes [2,6]. Subclinical TB can be detected using existing radiologic or microbiologic investigations. Subclinical TB is reported to comprise between one-fifth to one-half of cases among the bacteriologically positive patient population [7]. The current TB diagnostic cascade often relies on identifying symptoms, so that patients with subclinical TB may not be diagnosed.

Active case-finding (ACF) for TB is an important tool to find missing TB patients. Chest X-ray (CXR) is a tool that has been recommended by the World Health Organization (WHO) for screening people in ACF programs to find possible TB patients [8,9]. By using CXR within ACF, it is hypothesized that TB notification rates can be increased and cases can be identified and treated earlier.

Zero TB Yogyakarta (ZTB) is a collaboration between the Center of Tropical Medicine Faculty of Medicine, Public Health, and Nursing (FKKMK) Universitas Gadjah Mada, Dinas Kesehatan Yogyakarta Province, Indonesia and the Burnet Institute Melbourne Australia. The project, implemented in Yogyakarta commencing in 2020, aims to develop models of care for TB elimination through a comprehensive epidemic control approach (search—treat—prevent strategy) [10]. ZTB activities are implemented through community based ACF using mobile CXR, improved TB treatment through linkage of TB cases to treatment facilities, and provision of preventive therapy for TB infection in adults and children at high risk for TB. The aim of this study is to describe the spectrum of TB disease, including the yield of subclinical TB disease diagnosed through a mobile community based ACF program in Yogyakarta, and compare outcomes between groups of active TB disease defined by bacteriological and symptom status.

## 2. Materials and Methods

### 2.1. Design

This study was a sub-study embedded within the Zero TB Yogyakarta Program prospective observational cohort study.

### 2.2. Setting

This study was conducted in 2 districts of Yogyakarta Province, Indonesia reached by the Zero TB Yogyakarta mobile ACF program: Kulon progo (a rural area with estimated TB cases: 594–656 cases in 2023) and Yogyakarta City (an urban area with estimated TB cases: 1746–1928 in 2023 [11].

The ZTB ACF program operated a mobile van-based service including CXR facilities. Selection of screening sites was determined by the ZTB program together with the primary health center in the district, based on the highest case load of bacteriologically confirmed TB cases reported in the district over the preceding 3 years. Selected locations were chosen based on data from 21 primary health centers in Kulon progo district and 18 primary health centers in Kota Yogyakarta district. The locations of ACF screening were varied, and included village halls, primary health centers, schools, dormitories, prisons. People residing nearby to screening locations were invited for screening by the head of village or the primary health centers; no incentives were provided for participation.

### 2.3. Study Population

The study included people who attended and were eligible for TB screening in an ACF screening program in 2 districts, Kulon Progo and Yogyakarta Kota between 1 January 2021 to 30 June 2022. People were excluded from the study if they were less than 10 years old (as the spectrum of TB disease and outcomes in this population differ from adolescents and adults) people already taking TB treatment at the time of ACF screening, people that were pregnant at the time of screening (and not eligible for screening chest X-ray) or participants who did not have data entered on final diagnosis including microbiological results.

All eligible screening participants underwent CXR and symptom screening. The interpretation of CXR was performed initially by trained doctors in consultation with a radiologist and respiratory specialist physician. Subsequently, qXR software v1.3.0.69 (Qure.ai, Mumbai, India), an algorithm-based software to interpret the CXR [12], was used commencing May 2021 in Kulon progo, and October 2021 in Yogyakarta City. A qXR score > 0.5 was considered as screening positive. All people who screened positive for possible TB (cough for two weeks, or hemoptysis, or with a positive X-ray screening) were asked to provide one sputum sample on the spot which was transported to a laboratory for testing with Xpert MTB/RIF (Cepheid, Sunnyvale, CA, USA).

Bacteriologically confirmed TB refers to a positive molecular test (Xpert MTB/RIF), and would also include positive acid-fast bacilli microscopy or mycobacteriology culture if these tests were done. In this ACF project, Xpert MTB/RIF molecular testing was routinely performed on one sputum specimen, and microscopy or culture were rarely performed.

Clinical symptoms were defined as one or more of the following symptoms: cough of any duration, fever, night sweats and/or unintentional weight loss. A clinical diagnosis was made by considering CXR findings, symptoms, past history, contact history and lack of improvement in symptoms after treatment with broad spectrum antibiotics. Diagnosis was made through weekly case discussion between Primary Health Centre (Puskesmas) Doctor, ZTB field team doctors and nurses, respiratory consultants, and pediatrician reviewing all patient’s data (bacteriology, symptoms, and CXR).

We defined 4 groups representing the active TB disease spectrum based on clinical and bacteriological findings as follows: Group 1: symptomatic, bacteriologically confirmed TB; Group 2: symptomatic, not bacteriologically confirmed; Group 3: asymptomatic, bacteriologically confirmed TB; Group 4: asymptomatic, not bacteriologically confirmed. In this study, we defined subclinical TB as group 3 and group 4, which are asymptomatic and either bacteriologically confirmed or not.

Patients received treatment in the healthcare facility (Puskesmas or referral hospital) as is standard in the Indonesian TB program. A specific counselling and education session to deliver the diagnosis and treatment information was provided to people diagnosed with TB. Otherwise, treatment support was in accordance with national guidelines and provided through the National TB program. Patient’s data and treatment were recorded in each treating healthcare facility, and uploaded to the National Data TB Record “Sistem Informasi Tuberculosis/SITB”.

### 2.4. Data Management and Statistics

The ZTB project routinely recorded data using a REDCap electronic data capture tool hosted by Universitas Gadjah Mada [13]. Data variables collected include demographic data, symptoms, past history of TB, contact history of TB, risk factors including HIV status, smoking, alcohol and diabetes mellitus, chest X-ray findings, Xpert MTB/Rif results, TB diagnosis, and TB treatment outcome. The program had regular data review and data cleaning procedures.

For this study, data were exported from the ZTB REDCap database between 1 January 2021 and 30 June 2022. Treatment outcome data was also obtained from the national SITB database as censored at 8 February 2023. All the data were imported and analyzed in Stata version 17 (StataCorp LLC, College Station, TX, USA).

The yield of the TB ACF diagnostic cascade was described by counts and proportions. Variables were summarized with mean and standard deviation for continuous variables and count and percentage for categorical variables. Statistical associations were assessed with ANOVA, chi square and Fishers exact test as appropriate. Factors associated with successful treatment outcomes (age, sex, diabetes, HIV status, cavity on chest X-ray, symptom status (clinical vs. subclinical), bacteriological confirmation status) were analyzed using univariate and multivariate logistic regression analysis with *p*-value of 0.05 considered evidence of significance.

### 2.5. Ethics Approval

This study already obtained ethics approval from Medical and Health Research Ethics Committee, Faculty of Medicine, Public Health and Nursing, Universitas Gadjah Mada (Ref. No. KE/FK/1331/EC/2022), 24 October 2022. Permission to conduct the study was obtained from the Yogyakarta City and Kulon Progo District Health Office. Participants in the Zero TB Yogyakarta project were asked to sign an informed consent either to agree or disagree to screening.

## 3. Results

**Figure 1:**
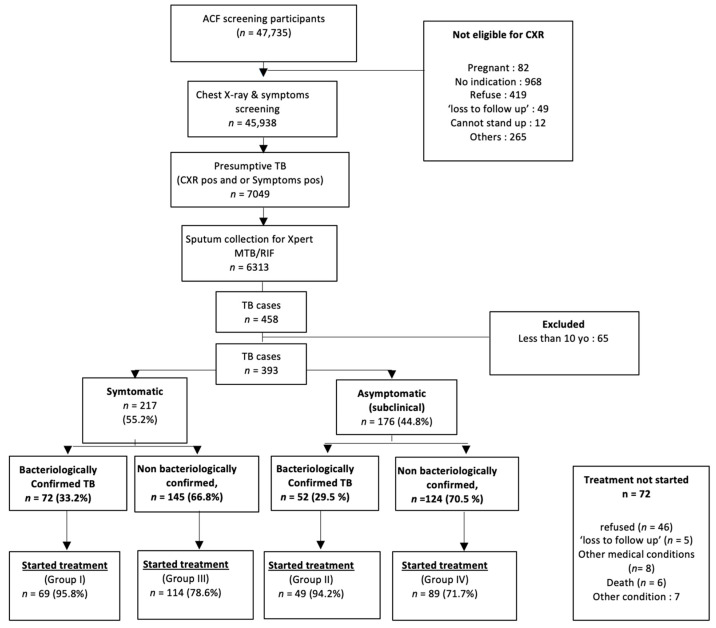
Diagnosis and treatment cascade from the Active Case Findings.

Between 1 January 2021 and 30 June 2022, 47,735 people attended the ACF program in two districts (Figure 1). The yield of TB disease within ACF in this study was 0.86% (393/45,938), 55.2% (217/393) were symptomatic and 44.8% (176/393) asymptomatic (subclinical TB). Among those who were symptomatic, the proportion of bacteriologically confirmed TB was 33.2%; the proportion of bacteriologically confirmed among asymptomatic (subclinical) TB was 29.5%. While among all bacteriologically confirmed cases (*n* = 124), the proportion of asymptomatic (subclinical) TB was 41.9% (Figure 1).

In total of 321 people with active TB, 68.2% were male; 2.5% were aged 10–18 years old; and 43.3% were >60 years old; 41.4% were underweight (BMI < 18.5); 10% had a history of TB treatment; and 10.3% were people living with HIV. A total of 7 (5.9%) rifampicin resistant cases were diagnosed amongst bacteriologically confirmed cases. There were significant differences between groups in age, screening location (rural vs. urban), previous TB treatment, HIV status, and cavity on X-ray (Table 1).

Pre-treatment loss to follow up (LTFU) was high with 18.3% (72/393) TB patients not starting treatment. The proportion not starting treatment amongst bacteriologically confirmed TB (4.8% 6/124), was much lower than non-bacteriologically confirmed TB (24.5% 66/269) (Figure 1).

Successful treatment outcome was highest in the symptomatic bacteriologically confirmed group (78.3% success), and lowest in the asymptomatic, bacteriologically negative group (62.9%). Overall proportions of LTFU during treatment were 9% and not evaluated 17.1% (55/321), and both were highest in the asymptomatic, bacteriologically negative group (20.4% LTFU and 23.6% not evaluated) (Table 2). Only 2 (28.6%) patients with rifampicin resistance had successful outcomes. In univariate analysis, the symptomatic group had a significantly lower odds of unsuccessful treatment (aOR 0.59, CI 0.36–0.96). When adjusting for other measured factors, in multivariate analysis there was weak evidence for a difference between symptomatic and subclinical TB being associated with an unsuccessful outcome (aOR 0.6, CI 0.36–0.998, *p* = 0.049) (Table 3).

## 4. Discussion

In this prospective study of the TB disease spectrum and treatment outcomes diagnosed through an active case finding program, we report substantially high proportions of subclinical TB, high pre-treatment LTFU, high on treatment LTFU and outcome not evaluated, and worse outcomes among subclinical TB patients.

A high proportion (41.9%) of bacteriologically confirmed TB were subclinical TB. These individuals diagnosed through CXR screening, could be missed by symptom screening and may not seek healthcare, resulting in continuing transmission in the communities. The proportion of subclinical TB among bacteriologically confirmed TB has been reported to range between 36.1% and 79.7% (median 50.4%) [7]. The proportion of subclinical TB may change according to the clinical criteria used in studies and also the sputum sample and bacteriological confirmation method. Our findings support the World Health Organization recommendations to include CXR in active TB case finding, as symptom screening alone will miss a high proportion of bacteriologically positive TB cases [8].

A large proportion of TB cases were diagnosed as bacteriologically negative and symptom negative TB cases in this study. Without bacteriological confirmation, this group could represent a mix of subclinical TB or of misdiagnosis. In Indonesia, only 56% of cases reported in 2021 were bacteriologically confirmed [14]. Even in TB centers utilizing more intensive diagnostic procedures diagnosing TB can be challenging, with culture negative TB reported to occur in 18.8% in a study in Peru [15]. Changes in diagnostic algorithms likely results in different proportions. This study only used one sputum sample for molecular diagnosis, and culture was not done in all patients. This could lower the proportion of those bacteriologically confirmed. Symptom screening in this study was done by trained ACF nurses. Although they were all trained, medical history taking is a skill and it is possible that in a busy screening program that symptoms are not elicited, that people don’t want to report symptoms during a screening or may not consider minor symptoms as relevant to report.

The spectrum of TB disease ranges from subclinical with no signs/symptoms, subclinical TB with unrecognized sign/symptoms, and TB disease with sign/symptoms [5]. Asymptomatic patients in our study had CXR abnormalities consistent with TB, however some had fibrosis, without symptoms and without TB treatment history. These findings could represent TB that is already eliminated, dormant or still active (sub-clinical) or fibrosis resulting from different pathology. Differentiating between these possibilities within a TB ACF program can be challenging. There is little guidance for TB ACF programs on further investigation or follow up for those with CXR abnormalities and no symptoms. The inclusion of young adolescents (aged 11–14 years) in this study may also contribute to the spectrum of disease in the study with a higher likelihood of having bacteriologically unconfirmed TB, however this was only a small proportion of the study population.

Pretreatment and on treatment LTFU and not evaluated were high in this population. The treatment success rate is lower than that reported by Indonesia in 2021 of 86%. However, it has been noted that there may be high rates of treatment not evaluated that is not included within the National treatment success calculations [16]. Pretreatment and on treatment LTFU/not evaluated were not evenly distributed in our study with the asymptomatic bacteriologically negative group having worse outcomes. This could potentially be due to beliefs in being healthy, misdiagnosis of CXR abnormalities, or less attention from healthcare workers to this group’s needs. Other studies have reported higher success rates among subclinical TB [17]. It is possible that drug resistance may have contributed to the low success rates, with rifampicin resistance among bacteriologically confirmed cases higher than the 2.2% reported amongst new TB cases in Indonesia.

Subclinical TB is likely to be more significant epidemiologically than has previously been recognized. Prevalent TB, especially those likely to be missed by passive case finding, includes a substantial proportion of subclinical TB, which even if less infectious is likely to contribute substantially to transmission due to the large numbers and prolonged periods without diagnosis [18]. Learning how to improve ACF, including linkage to care and ensuring appropriate support to achieve completion of treatment is therefore vital for achieving the END-TB strategy goals in Indonesia. However, it is also important that consideration is given to the cost-effectiveness of different algorithms for investigating people who screen positive on ACF and are negative with a single sputum test. As ACF programs scale up the potential numbers of people with possible TB will be high, and the programmatic costs and resources for further investigation and follow up need to be considered in ACF programs.

A key strength of this study is the prospective design with routine data quality cleaning and combined data collected from the ACF program and the national recording data system on TB (SITB). Limitations of this study include the proportion of missing data for outcomes, categorized as not evaluated by WHO, even after multiple attempts to identify missing data. This could potentially under-estimate successful treatment outcomes as treatment may be completed but not being recorded in the national data record, or patients being transferred to a different facility. Equally this could conceal higher death or failure rates. The COVID-19 pandemic, and the change from one national TB reporting database to a new TB database, which both occurred during the time-period of this study, may have contributed to worse reporting of treatment outcomes. Secondly, the final diagnosis was made within the routine program, by different doctors at different facilities with varying TB expertise. Not all the asymptomatic patients, specifically the bacteriologically negative group, have access to the referral hospital to get further follow up to establish the diagnosis. Additionally, limitations of the national insurance referral system did not allow referral or cost coverage for some patient groups. Also, the change in CXR reading methodology during the study period from doctor reading to CAD software may also have had an impact which we were not able to assess. These factors could have contributed to either an increase in missed diagnosis or over diagnosis. However, all doctors had access to discussion with pulmonary specialist, which would help with consistency of the approach for challenging diagnostic cases.

Our findings emphasized the importance of using CXR for ACF programs [8]. A higher proportion of bacteriologically negative and symptom negative patients should be anticipated when scaling up ACF programs that include CXR in Indonesia. In addition, it is important that ACF programs ensure a strong referral linkage system to provide access to further diagnostic investigation for this group, and also ensure support for treatment. Further follow up is needed of the cost-effectiveness of CXR based ACF in Indonesia, and to look at the not evaluated treatment outcomes within the TB program to identify the reasons why this is occurring. Finally, community-based TB active case finding interventions have shown mixed results and further evidence on their effectiveness is needed [19,20].

## 5. Conclusions

In this ACF program utilising CXR, the spectrum of TB disease included high proportions of subclinical TB and of bacteriologically negative TB. Successful outcomes for TB treatment were lower among subclinical, bacteriologically unconfirmed TB cases. Efforts to improve linkage to care and national reporting are needed when implementing TB ACF programs.

## Figures and Tables

**Table 1:** Baseline characteristic of people starting TB treatment by TB disease groups.

		Total	Symptom (+) & Bacteriology (+)	Symptom (+) & Bacteriology (−)	Symptom (−) & Bacteriology (+)	Symptom (−) & Bacteriology (−)	*p*-Value
		*n* = 321	*n* = 69	*n* = 114	*n* = 49	*n* = 89	
Sex	Male	219 (68.2%)	45 (65.2%)	76 (66.7%)	38 (77.6%)	60 (67.4%)	0.49
	Female	102 (31.8%)	24 (34.8%)	38 (33.3%)	11 (22.4%)	29 (32.6%)	
Age		52.9 (18.7)	44.9 (17.6)	56.0 (18.4)	52.9 (17.3)	55.1 (19.2)	<0.001
Age Group	10–<18	8 (2.5%)	2 (2.9%)	2 (1.8%)	0 (0.0%)	4 (4.5%)	<0.001
	18–<60	174 (54.2%)	53 (76.8%)	49 (43.0%)	29 (59.2%)	43 (48.3%)	
	≥60	139 (43.3%)	14 (20.3%)	63 (55.3%)	20 (40.8%)	42 (47.2%)	
Screening location	Rural	112 (37.8%)	21 (33.3%)	51 (49.5%)	8 (16.7%)	32 (39.0%)	0.001
	Urban	184 (62.2%)	42 (66.7%)	52 (50.5%)	40 (83.3%)	50 (61.0%)	
Nutritional Status	BMI ≥18.5 to <23	120 (37.4%)	27 (39.1%)	36 (31.6%)	17 (34.7%)	40 (44.9%)	0.28
	BMI < 18.5	133 (41.4%)	29 (42.0%)	55 (48.2%)	17 (34.7%)	32 (36.0%)	
	BMI > 23	68 (21.2%)	13 (18.8%)	23 (20.2%)	15 (30.6%)	17 (19.1%)	
Previous TB treatment	New	280 (90.0%)	54 (81.8%)	104 (94.5%)	39 (83.0%)	83 (94.3%)	0.008
	Previously treated	31 (10.0%)	12 (18.2%)	6 (5.5%)	8 (17.0%)	5 (5.7%)	
Smoking Status	Never smoked	165 (51.4%)	34 (49.3%)	57 (50.0%)	26 (53.1%)	48 (53.9%)	0.92
	Current or previously smoked	156 (48.6%)	35 (50.7%)	57 (50.0%)	23 (46.9%)	41 (46.1%)	
HIV Status	Negative or unknown	288 (89.7%)	66 (95.7%)	96 (84.2%)	48 (98.0%)	78 (87.6%)	0.015
	Positive	33 (10.3%)	3 (4.3%)	18 (15.8%)	1 (2.0%)	11 (12.4%)	
Diabetes Mellitus Status	Negative or unknown	288 (89.7%)	60 (87.0%)	104 (91.2%)	41 (83.7%)	83 (93.3%)	0.26
	Positive	33 (10.3%)	9 (13.0%)	10 (8.8%)	8 (16.3%)	6 (6.7%)	
Cavity on Chest X-ray	no cavity	273 (85.0%)	51 (73.9%)	101 (88.6%)	44 (89.8%)	77 (86.5%)	0.031
	cavity	48 (15.0%)	18 (26.1%)	13 (11.4%)	5 (10.2%)	12 (13.5%)	
Rifampicin resistance	Rifampicin susceptible	110 (93.2%)	65 (94.2%)		45 (91.8%)		0.49
	Rifampicin resistant	7 (5.9%)	3 (4.4%)		4 (8.2%)		
	Indeterminate or missing	1 (0.8%)	1 (1.4%)		0 (0%)		

**Table 2:** Treatment Outcome in Active TB Group.

		Total	Symptom (+) & Bacteriology (+)	Symptom (+) & Bacteriology (−)	Symptom (−) & Bacteriology (+)	Symptom (−) & Bacteriology (−)
		*n* = 321	*n* = 69	*n* = 114	*n* = 49	*n* = 89
Treatment outcome	successful	227 (70.7%)	54 (78.3%)	84 (73.7%)	33 (67.3%)	56 (62.9%)
	Unsuccessful	94 (29.3%)	15 (21.7%)	30 (26.3%)	16 (32.7%)	33 (37.1%)
Treatment outcome (detailed)	cure	76 (23.7%)	42 (60.9%)	0 (0.0%)	30 (61.2%)	0 (0.0%)
	completed	151 (47.0%)	12 (17.4%)	84 (73.7%)	3 (6.1%)	56 (62.9%)
	LTFU	29 (9%)	5 (7.2%)	12 (10.5%)	3 (6.1%)	9 (10.1%)
	failure	2 (0.6%)	0 (0.0%)	0 (0.0%)	2 (4.1%)	0 (0.0%)
	death	8 (2.5%)	2 (2.9%)	2 (1.8%)	1 (2.0%)	3 (3.4%)
	Not evaluated	55 (17.1%)	8 (11.6%)	16 (14.0%)	10 (20.4%)	21 (23.6%)

**Table 3:** Univariate and multivariate analysis on factors that affect treatment outcome.

		Total	Successful	Unsuccessful	Odds Ratio	95% CI	Odds Ratio	95% CI
		*n* = 321	*n* = 227	*n* = 94				
Age		52.9 (18.7%)	51.1 (19.0%)	57.2 (17.4%)	1.02	(1.00, 1.03)	1.01	[1.00, 1.03]
Sex	Male	219 (68.2%)	160 (70.5%)	59 (62.8%)	Reference		Reference	
	Female	102 (31.8%)	67 (29.5%)	35 (37.2%)	1.42	(0.85, 2.35)	1.29	[0.75, 2.22]
Nutritional Status	BMI ≥ 18.5 to <23	120 (37.4%)	82 (36.1%)	38 (40.4%)	Reference		Reference	
	BMI < 18.5	133 (41.4%)	96 (42.3%)	37 (39.4%)	0.83	(0.48, 1.43)	0.83	[0.47, 1.48]
	BMI > 23	68 (21.2%)	49 (21.6%)	19 (20.2%)	0.84	(0.43, 1.61)	0.85	[0.43, 1.70]
Previous TB treatment	New	280 (90.0%)	195 (88.6%)	85 (93.4%)	Reference		Reference	
	Previously treated	31 (10.0%)	25 (11.4%)	6 (6.6%)	0.55	(0.22, 1.39)	0.63	[0.24, 1.66]
HIV Status	Negative or unknown	288 (89.7%)	199 (87.7%)	89 (94.7%)	Reference		Reference	
	Positive	33 (10.3%)	28 (12.3%)	5 (5.3%)	0.40	(0.15, 1.07)	0.44	[0.13, 1.47]
Diabetes Mellitus Status	Negative or unknown	288 (89.7%)	207 (91.2%)	81 (86.2%)	Reference		Reference	
	Positive	33 (10.3%)	20 (8.8%)	13 (13.8%)	1.66	(0.79, 3.50)	1.40	[0.63, 3.13]
Cavity on Chest X-ray	no cavity	273 (85.0%)	193 (85.0%)	80 (85.1%)	Reference		Reference	
	cavity	48 (15.0%)	34 (15.0%)	14 (14.9%)	0.99	(0.51, 1.95)	1.11	[0.54, 2.28]
Symptom status	Asymptomatic	138 (43.0%)	89 (39.2%)	49 (52.1%)	Reference		Reference	
	Symptomatic	183 (57.0%)	138 (60.8%)	45 (47.9%)	0.59	(0.36, 0.96)	0.60	[0.36, 1.00]
Bacteriological Confirmation	Bacteriologically negative	203 (63.2%)	140 (61.7%)	63 (67.0%)	Reference		Reference	
	Bacteriologically positive	118 (36.8%)	87 (38.3%)	31 (33.0%)	0.79	(0.48, 1.31)	0.84	[0.48, 1.48]

## Data Availability

Due to data privacy concerns, data is not made publicly available. However, reasonable data requests may be granted through contacting the corresponding author.
